# Prediction and Prevention of Delayed Post-polypectomy-induced Ulcer Hemorrhage Based Upon an Anatomic Study and Multivariable Analyses

**DOI:** 10.1007/s10620-026-09710-2

**Published:** 2026-02-10

**Authors:** Dennis M. Jensen, Jeffrey Gornbein, Phillip Fejleh, Michael Lewis, Hanlin Wang

**Affiliations:** 1 David Geffen School of Medicine at UCLA, Los Angeles, CA USA; 2 GI Hemostasis Research Unit, Division of Digestive Diseases, Department of Medicine, VA Greater Los Angeles Healthcare System, Building 115, Room 318, 11301 Wilshire Blvd, Los Angeles, CA 90073-1003 USA; 3 Vatache and Tamar Manoukian Division of Digestive Diseases, Department of Medicine, Ronald Reagan UCLA Medical Center, Los Angeles, CA USA; 4 Statistics Core, UCLA Medical Center, Department of Medicine, Los Angeles, CA USA; 5 Kaiser Permanente San Diego Zion and San Diego Medical Center, San Diego, CA USA; 6 Department of Anatomic Pathology, VA Greater Los Angeles Healthcare System, Los Angeles, CA USA; 7 Department of Anatomic Pathology, Ronald Reagan UCLA Medical Center, Los Angeles, CA USA

**Keywords:** Delayed post-polypectomy hemorrhage, Empiric hemoclip closure, Blood flow monitoring, Doppler endoscopic probe

## Abstract

**Background:**

Better ways to predict and prevent delayed post-polypectomy-induced ulcer hemorrhage (DPPIUH) are needed.

**Aims:**

(1) To correlate Doppler endoscopic probe (DEP) detection of PPIU blood flow with polyp artery size on histology. (2) To report risk factors which predict DPPIUH and prevent it.

**Methods:**

96 high-risk patients with benign colon polyps and 10–40 mm PPIUs were included in this cohort study which analyzed prospectively collected data. Coded polyps were analyzed by expert GI pathologists to report artery size at resection margins. 28 patients with severe DPPIUH’s all DEP positive (+) for arterial blood flow including 15 with prior empiric hemoclip (HC) closure were compared with 68 similar high-risk patients without DPPIUHs: 34 DEP+ treated to obliterate blood flow; 22 DEP negative not treated; and 12 DEP+ not treated. 16 potential risk factors for DPPIUH were simultaneously assessed using multivariable logistic analysis and a classification tree model.

**Results:**

The odds ratio (95% CI’s) of DEP positivity predicting medium or large polyp arteries was 23.4 (5.73, 11.0)—*p* < 0.001. Risk factors predicting DPPIUH were DEP positivity, medium, or large size polyp artery, right colon polyp location, and empiric HC closure. DEP-guided treatment predicted the absence of DPPIUH.

**Conclusions:**

(1) DEP positivity and medium or large artery size on polyp histology highly correlated and were new risk factors predicting DPPIUH. (2) Other significant risk factors predicting DPPIUH were right colon polyp location and empiric PPIU HC closure. (3) DEP-guided treatment prevented DPPIUH.

## Introduction

Delayed post-polypectomy-induced ulcer hemorrhage (DPPIUH) is an increasingly common and potentially serious complication of colonoscopy [1, 2]. The incidence ranges from a low of 0.44% of colon polypectomy cases in a national database study to 6.5% for polyps ≥ 20 mm [1, 2]. For patients with co-morbidities and chronic anti-thrombotic medication use, the incidence is reported to exceed 11% [3]. The best ways to predict and prevent it have not been established. No medical treatment has been reported to heal PPIU’s or prevent DPPIU. Empiric through-the-scope hemoclip (HC) closure of PPIUs is often advocated by expert colonoscopists and interventional endoscopists for polyps 20 mm or larger [4–8], but this treatment is ineffective in the left colon or rectum, it utilizes multiple HCs, often incompletely closes PPIUs, and is expensive [9–11]. Also, single-layer HC closure of the mucosal layer buries submucosal vessels often without obliterating arterial blood flow [12, 13]. After HCs slough off, submucosal arteries in the PPIU are exposed and DPPIUH can occur [12, 13]. For high-risk patients with co-morbidities and polyps and/or PPIUs between 10 and 19 mm in size, the risk factors for DPPIUH, its prediction, and its prevention have not been determined [13]. In recent reports, the PPIU size after EMR or thermal coagulation of pedunculated polyps was more predictive of delayed hemorrhage than the polyp size [12, 13]. Anatomic studies, better treatments, and prediction models are needed to advance our knowledge about DPPIUH and to improve prophylactic treatment. This is especially important in high-risk patients who are at increased risk for this common and potentially serious complication of routine colonoscopy with polypectomy [1–3].

Our hypotheses are that arterial blood flow detection in PPIUs and polyp artery size at the resection margins will be new important predictors of DPPIUH and are the anatomic and physiologic basis of DPPIUH. Furthermore, focal treatment of arteries in PPIU’s will improve prophylactic colonoscopic treatment for prevention of DPPIUH. These hypotheses are based in part upon our previous reports of DEP monitoring of arterial blood flow detection in peptic ulcer bleeds (PUBs) to improve diagnosis, risk stratification, and treatment of patients with severe non-variceal upper GI hemorrhage [14, 15].

The specific aims of our study were (1) to correlate Doppler endoscopic probe (DEP) detection of arterial blood flow in PPIUs after polyp resection with artery size at the resection margin on histopathology; (2) To report risk factors which predict DPPIUH and prevent it; and (3) To compare the effectiveness of empiric HC closure with DEP-guided treatment for prevention of DPPIUH.

## Methods

After Institution Review Board (IRB) approvals, 96 high-risk patients with PPIUs 10–40 mm and benign colon polyps resected during outpatient colonoscopies for screening or surveillance were investigated. This was a cohort study with analysis of prospectively collected data for patients who had DEP monitoring of PPIUs, *benign polyps,* and were followed for 30 days after colon polypectomy.

All polyps were removed with endoscopic mucosal resection (EMR) if sessile and/or thermal coagulation if pedunculated. All patients were required to have DEP monitoring of arterial blood flow in PPIUs during the index colonoscopy and histologically evaluable polyps for malignancy. Malignant appearing polyps such as firm sessile lesions with ulcerations or those that could not be raised up by submucosal injection were excluded during colonoscopy and did not have DEP monitoring. All cases in this observational study had DEP monitoring, whether this was used as a guide for treatment or not. This was not a DEP-guided interventional study nor an RCT. The choice of colonoscopic treatment or not was the colonoscopist’s and based upon their experience, procedure time available, and convenience.

**Fig. 1:**
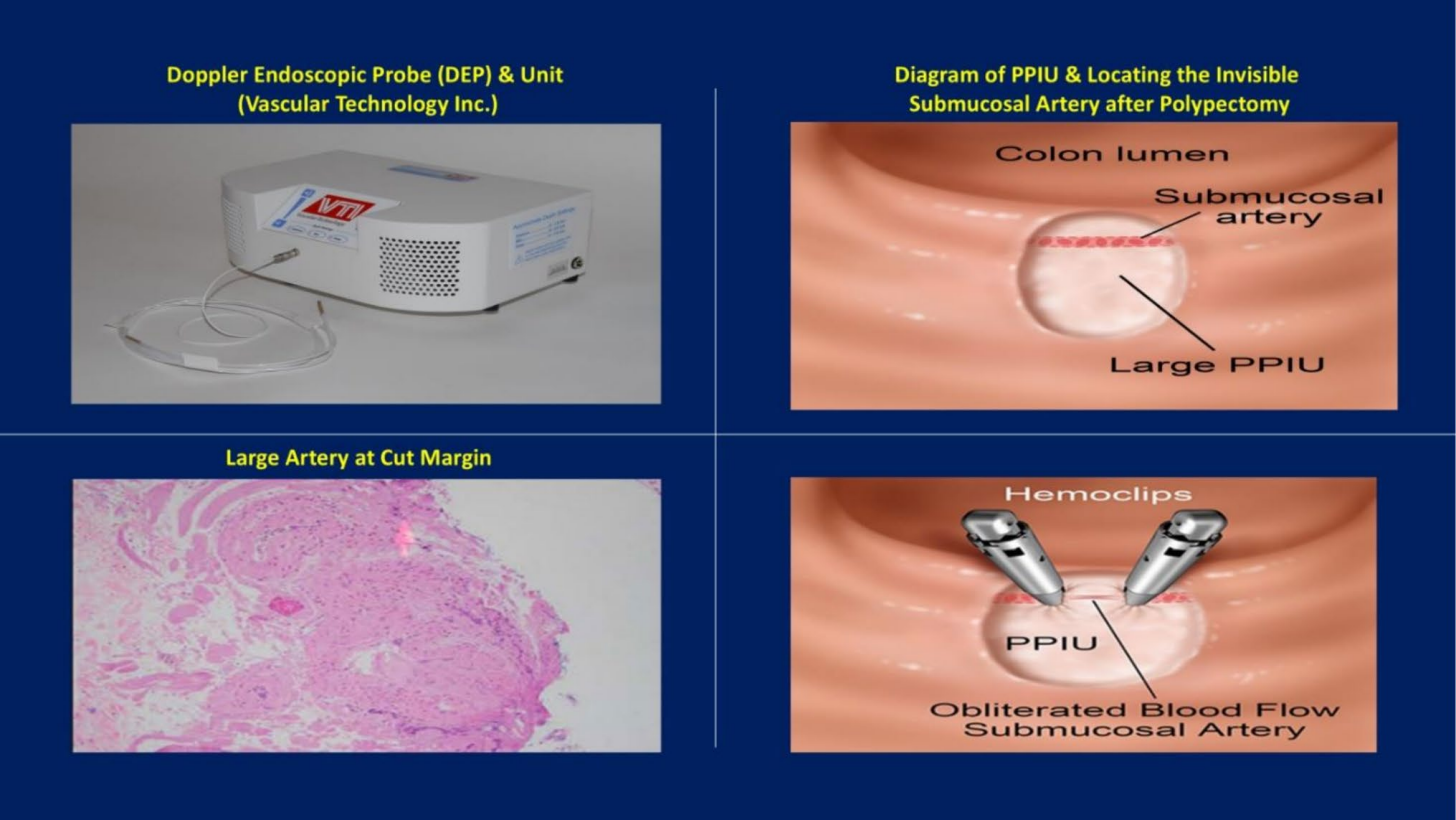
Results of interrogating the base of a post-polypectomy ulcer with Doppler endoscopic probe to detect arterial blood flow and map a large invisible submucosal artery

See Fig. 1 which illustrates DEP interrogation of a PPIU to locate and map an invisible submucosal artery and treat it after polypectomy. In patients with DEP-guided treatment, 2–3 hemoclips were deployed along the submucosal artery in the PPIU to obliterate blood flow as documented by repeat DEP monitoring after this treatment.

Exclusions were malignant polyps, PPIUs smaller than 10 mm or larger than 40 mm, and polyps which were not evaluable for histological diagnosis to exclude malignancy or had malignancy on removal. Severe DPPIUH was defined as hospitalization for severe hematochezia after colon polypectomy diagnosed by repeat colonoscopy based upon stigmata of recent hemorrhage (SRH) in a PPIU [16].

High-risk clinical features for delayed PPIUH that are reported include age over 65 years, high co-morbidity scores (e.g., Charlson or American Society for Anesthesiology—ASA—scores), and chronic use of anti-thrombotic medications (e.g., anti-coagulants and/or anti-platelet drugs). High-risk anatomic features include polyp configuration (e.g., sessile vs. pedunculated), large polyp size (20 mm or greater), and right colon location of polyps [4–8, 17–19].

When DEP was utilized for mapping submucosal arteries in PPIUs after the polypectomy, the arteries coursed straight across the PPIU instead of being serpiginous, as shown in Fig. 1. For focal treatment guided by DEP, 2–3 hemoclips were deployed along the artery to obliterate blood flow. This contrasted with many more hemoclips for empiric closure of PPIUs (e.g., 6–8 HC to close 15–40 mm PPIUs).

**Fig. 2:**
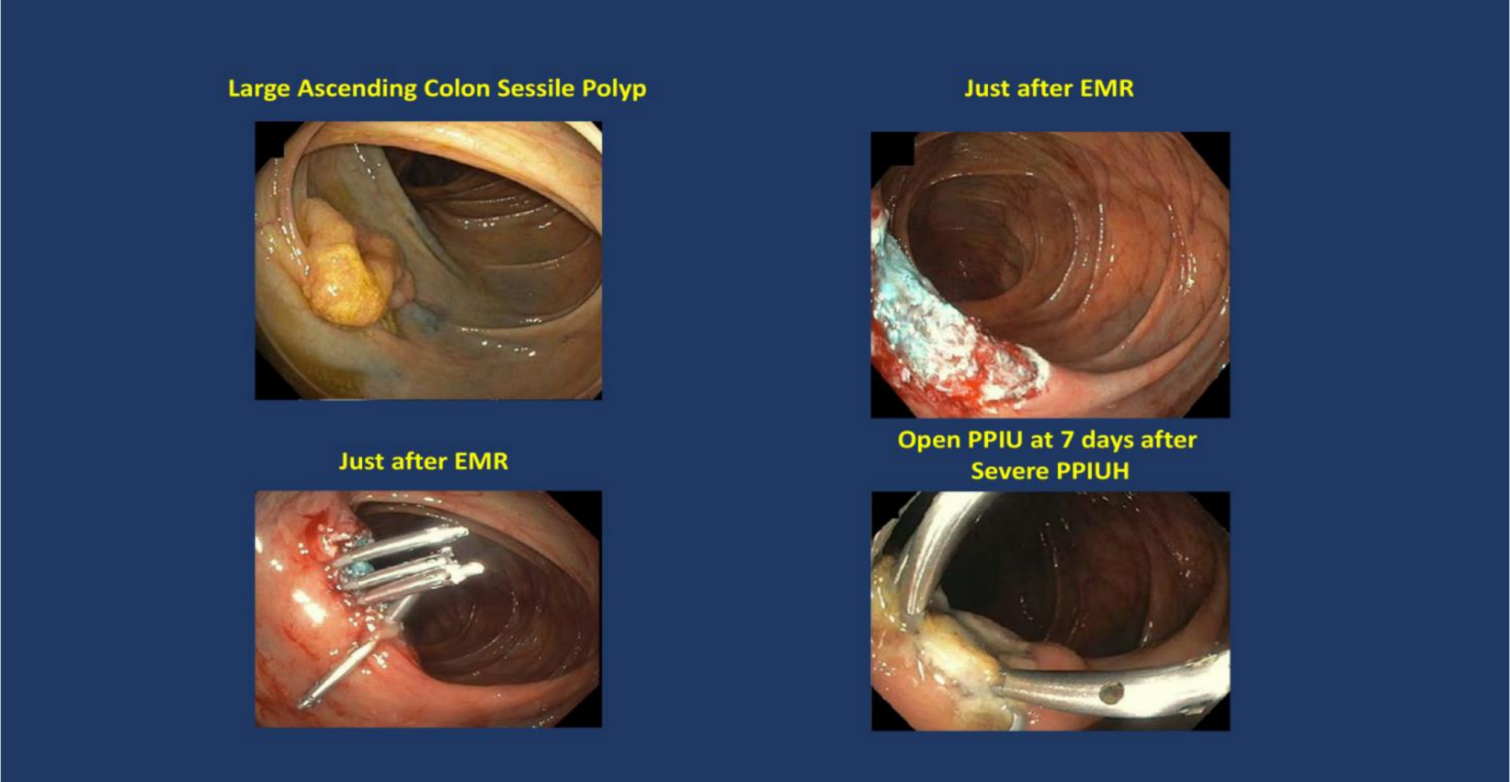
Patient with empiric hemoclip closure with multiple hemoclips of a 20-mm post-polypectomy ulcer but who developed delayed severe hemorrhage after 5 days. Shown are the findings of an open post-polypectomy ulcer with 4 hemoclips remaining when urgent colonoscopy after hospitalization for severe hematochezia

**Fig. 3:**
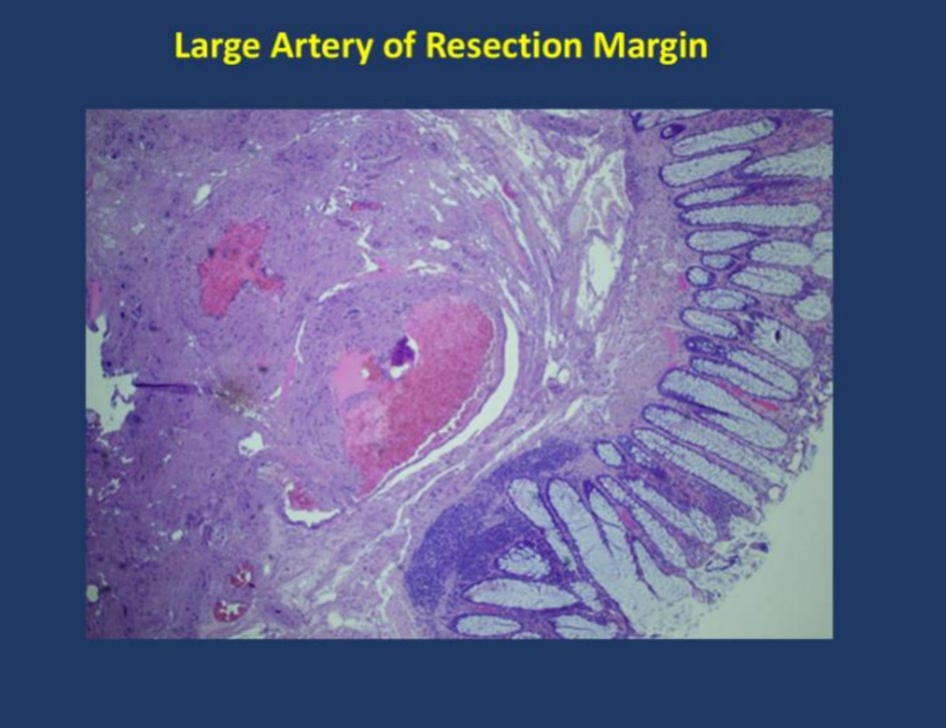
A large artery was documented at the polyp resection margin of the patient described in Fig. 2

When patients were hospitalized after index colonoscopy with severe hematochezia from DPPIUH and had urgent colonoscopy, we identified PPIUs with SRH (including active bleeding, non-bleeding visible vessel—NBVV, an adherent clot, or a flat spot) and over 90% had positive arterial blood flow underneath the SRH by DEP monitoring [13, 16]. In those who had prior empiric hemoclip closure during the index colonoscopy, several hemoclips had fallen off the mucosa and the PPIUs were open, exposing a submucosal artery with SRH. Refer to Fig. 2 as an example of the open PPIU on urgent colonoscopy after delayed PPIUH and Fig. 3 for the histologic findings of the artery at the resection margin of the index polypectomy in this patient.

Coded polyp specimens were analyzed by expert GI pathologists (M. Lewis MD and H. Wang, MD, PhD) without knowledge of the DEP findings or whether delayed PPIUH had occurred. They reported the polyp histologic diagnosis and the presence or absence of arteries and size of blood vessels at the resection margin. Arterial blood flow findings in PPIU’s were correlated with artery size at the polyp resection margin.

### Statistical Methods

Bivariate analysis. The *p* values for comparing categorical variables for delayed bleeding or for assessing the association between two categorical variables were computed using Fisher’s exact test. Odds ratios (OR) are also reported.

The p values for comparing continuous variables such as polyp size were computed using the non-parametric Wilcoxon rank-sum test since the continuous variables did not usually follow a normal distribution.

Multivariable analysis. Both classification tree (binary recursive partition) and logistic regression models were explored to assess the simultaneous association of the 16 potential predictors for delayed bleeding. Classification tree results are reported since these were the most accurate. Sensitivity, specificity, and accuracy defined as accuracy = (sensitivity + specificity)/2 are reported where accuracy was maximum. In addition, the receiver operator curve (ROC) area, which is also the concordance (C) statistic, is reported as an additional measure of accuracy. Both nominal sensitivity, specificity, and accuracy as well as the corresponding fivefold cross-validated values are given.

Computations were carried out using R 4.3.2 (R Foundation for Statistical Computing, Vienna, Austria, https://www.R-project.org/).

## Results

This study included 96 patients with a PPIU between 10 and 40 mm, 28 patients with severe DPPIUH, all DEP positive for arterial blood flow detected in PPIUs. Fifteen of these patients with DPPIUH had prior empiric HC closure during the index colonoscopy and 13 did not have HC closure. The median time to presentation with severe delay PPIUH was 4 days after the index colonoscopy. The 28 patients with DPPIUH were compared with 68 similar high-risk patients without DPPIUHs including the first subgroup of 34 patients who were DEP positive on arterial blood flow monitoring after polypectomy and had focal treatment with hemoclips guided by DEP and obliteration of arterial blood flow during the index colonoscopy, as shown in Fig. 1. Two other subgroups without colonoscopic treatment during the index colonoscopy and with no DPPIUH were 22 DEP negative patients and 12 other patients with DEP positivity.

There were no major complications of the colonoscopy, polypectomy, or treatments (empiric hemoclip closure or DEP-guided treatments) such as perforations, post-coagulation syndrome, ileus, or abdominal pain. However, all the patients who developed severe delayed PPIU hemorrhage including those with empiric hemoclip closure were hospitalized.

**Fig. 4:**
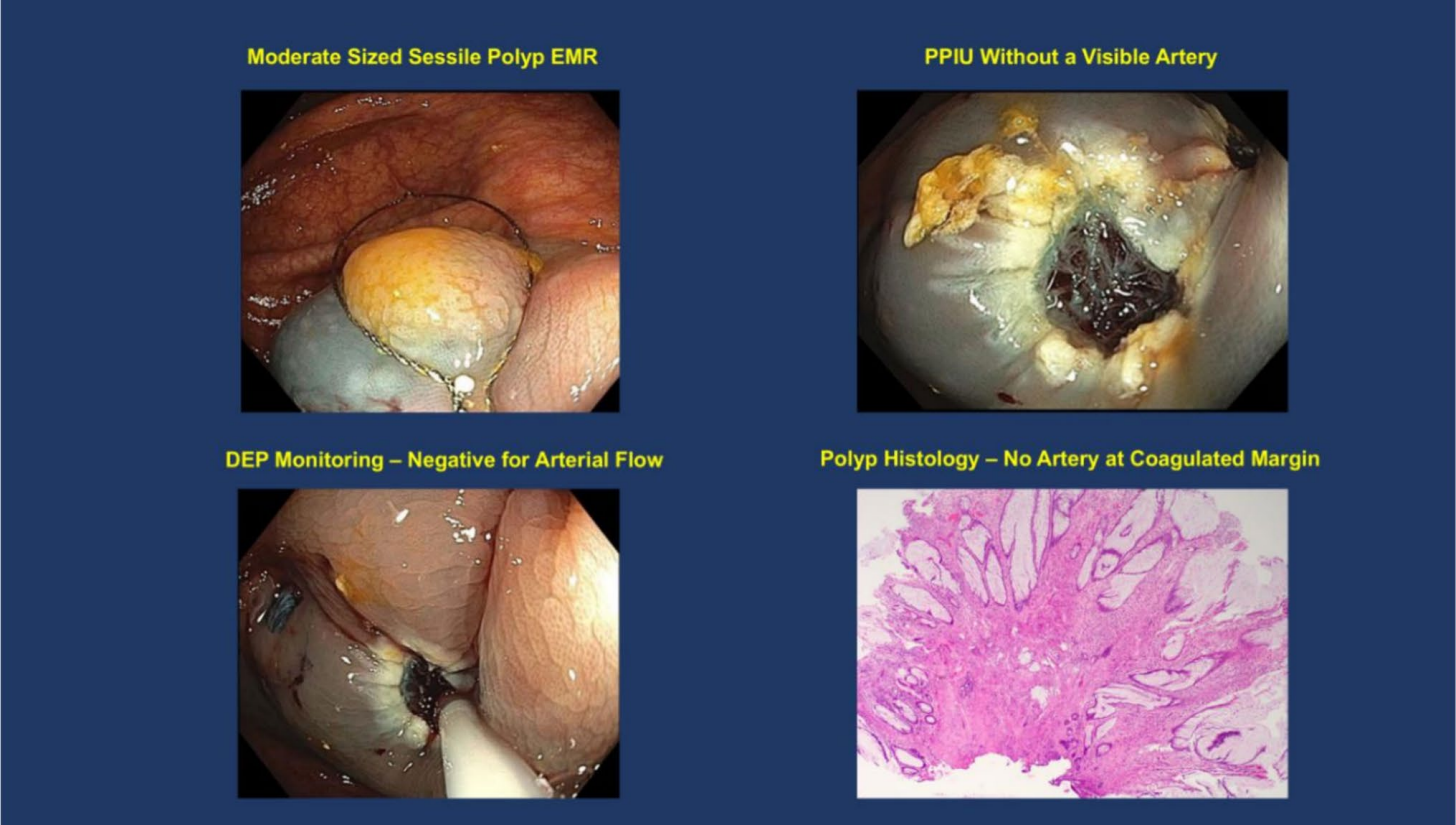
Resection margin of a benign polyp with a small artery reported in a patient with no arterial blood flow detected by Doppler probe. The patient had no endoscopic treatment and did not have delayed bleeding

In 22.9% (22/96) of the patients with PPIUs between 10 and 40 mm, no arterial blood flow was detected in PPIUs just after polypectomy. None had colonoscopic treatment and none developed DPPIUH. Eighty-one percent of these patients with no blood flow detected by DEP had small or no arteries on histopathology at the polyp resection margin. See Fig. 4 as a representative example.

In Table 1, the bivariate analysis results are shown comparing patients who developed DPPIUH with those who did not. Significant risk factors associated with DPPIUH were DEP positivity, empiric HC closure, location of the polyp in the right colon (vs. left colon or rectum), polyp histology group, medium or large artery size (vs. small or none), and the combined predictor of DEP negativity and no or small artery (vs. DEP positivity and medium or large artery). Risks predicting absence of DPPIUH were DEP-guided HC treatment, no or small arteries at the polyp resection margin, and negative arterial blood flow on DEP monitoring. Some other clinical or colonoscopic risk factors reported by others to be predictive of DPPIUH were not significant in this study. These included anti-platelet use, anti-coagulants, polyp configuration (sessile vs. pedunculated), high ASA or Charlson morbidity scores, or large polyp or PPIU size [4–8, 17–19].

**Table 1:** Patient demographic, clinical, colonoscopic, and polyp results

	Delayed PPIU bleeding	No PPIU bleed	Bivariate *P* value
Number of patients	28	68	
Gender (female/male)	2/26	5/63	0.9999
Age (years)^†^	65.3 ± 10.9	68.1 ± 8.5	0.472
Race			0.988
African American	8	20	
Asian	1	4	
Caucasian	15	32	
Hispanic	4	11	
Native American	0	1	
Anti-platelet drugs/total	11/28	42/68	0.9999
Anti-coagulant drugs/total	9/28	20/68	0.8103
ASA score^†^	2.5 ± 0.74	2.3 ± 0.57	0.2575
Charlson score^†^	6.6 ± 2.2	6.3 ± 2.1	0.3102
Polyp location (right/left)	22/6	33/68	0.0072
Polyp type (sessile/pedunculated)	22/6	56/12	0.393
Polyp size^†^	18.5 ± 10	15.5 ± 6.6	0.2275
PPIU size^†^	17.4 ± 7.8	15.7 ± 5.4	0.3331
DEP positive/total	28/28	46/68	0.0003
Empiric HC closure/total	15/28	0/68	0.0000
DEP-guided treatment/total	0/28	34/68	0.0000
Polyp artery size (none or small/medium or large)‡	2/23	25/36	0.0041

	Delayed PPIU bleeding	No PPIU bleed	Bivariate *P* value
Polyp histology			0.0174
1. Hyperplastic	0	7	
2. Serrated adenoma	2	10	
3. Tubular adenoma	9	32	
4. Villous adenoma	18	17	
5. Inflammatory	0	1	
Histo polyp subtype^§^			0.0508
Others	2	18	
TA or TV	26	50	

Predictor^¶^		No	Yes	Total	Bleed rate (%)	*p* value
DEP negative and artery none-small		17	0	17	0.00	0.0023
DEP positive and/or medium-large artery		51	28	70	35.4	
	Totals	68	28	96	29.3	

^†^Mean ± Standard Deviation

^‡^On histopathology adequate submucosa of polyps was not included in 10.7% (3/28) with DPPIUH and 10.2% (7/68) of those with no DPPIUH so the artery size could not be evaluated

^§^Refer to the numbers listed (1–5) in the polyp histology row. Tubular adenomas or villous adenomas accounted for 79.2% all polyps

^¶^The last predictor is a combined one of DEP result (negative or positive) and polyp artery size (none-small vs. medium or large)

**Fig. 5:**
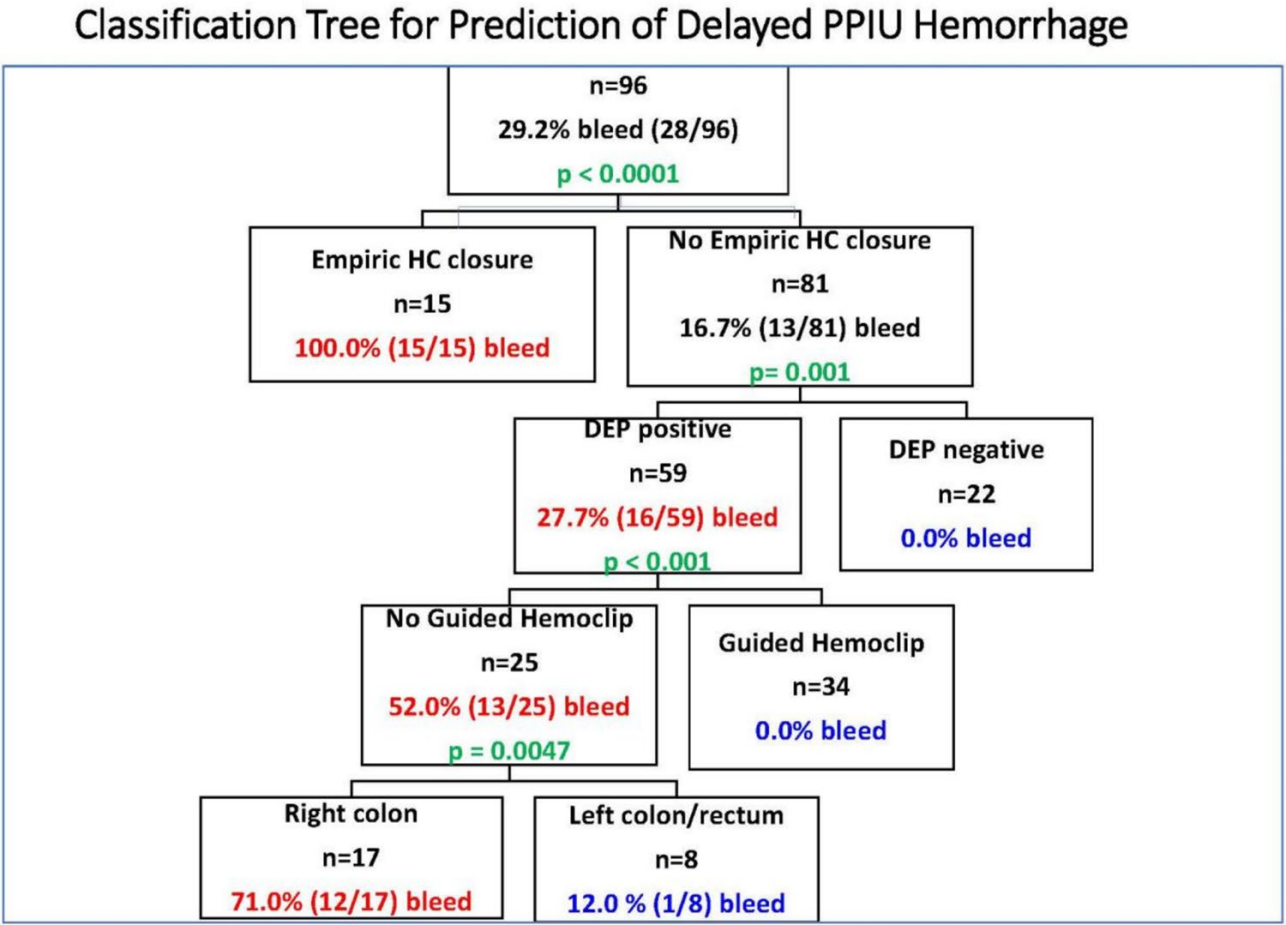
Classification tree results which can be utilized clinically to stratify patients by risk and for prevention of delayed post-polypectomy-induced hemorrhage. Listed are percents (and numbers) with delayed PPIU hemorrhage

In the multivariable classification tree modeling, risk factors predicting DPPIUH were empiric HC closure of PPIUs, DEP positivity, right vs. left colon or rectum location of polyps, and medium or large artery size in the polyp resection margin. DEP-guided treatment and no or small artery at polyp resection margin predicted the absence of DPPIUH. Similar to the bivariate analysis, other previously reported risk factors were not predictive of DPPIUH, as described above. The classification tree with the best results is shown in Fig. 5. The classification tree nominal sensitivity, specificity, and accuracy were 92.6%, 96.4%, and 94.5%, respectively. Cross-validation accuracy was 86.0%. The nominal C statistic was C = 0.980 and the cross validated *C* = 0.932. As shown in classification tree (Fig. 5), the most important predictor of delayed PPIU bleeding in patients with DEP positivity was empiric HC closure. For those who did not have empiric HC closure and were DEP negative, none had PPIU bleeding. Also other patients without bleeding were DEP positive who had DEP-guided hemocliping instead of empiric HC closure. Others without empiric HC closure of PPIUs in the right colon who were DEP positive but had no DEP-guided treatment developed delayed bleeding, whereas similar patients who had polyps in the left colon or rectum did not have PPIU bleeding.

The odds ratio (and 95% CIs) of DEP positivity predicting medium or large polyp arteries was 23.4 (5.73, 111.0)—*p* < 0.001. See Table 2.

**Table 2:** Correlation of DEP detection of arterial blood flow in PPIUs with artery size in polyp resection margin

Artery size on histology					
Variable	Level	None—small	Medium—large	Total	Medium or large (%)
DEP	Negative	17	4	21	19.0
DEP	Positive	10	55	65	84.6
	Total	27	59	86	
	pct DEP positive	37.0%	93.2%	75.6%	
	odds DEP positive	0.59	13.75	3.10	
		Odds ratio (OR)	23.4	Odds of DEP positive in medium-large vs. non-small artery	
		95% CI’s	(5.73; 110.0)	P < 0.001	

*DEP* Doppler endoscopic probe

Positive is detection of arterial blood flow in the PPIU and Negative is no detection

*For artery size on histology

**CI’s are 95% confidence intervals. On histopathology adequate submucosa of polyps was not included in 10.7% (3/28) with DPPIUH and 10.2% (7/68) of those with no DPPIUH so the artery size could not be evaluated

The relative direct costs of DEP-guided treatment to prevent DPPIUH were estimated to be significantly less than empiric hemoclip closure of PPIUs. This is based upon the following. The market price of a single disposable hemoclip and DEP catheter are similar, but only one DEP catheter is used in blood flow monitoring along with 2–3 hemoclips in PPIUs of 10–40 mm for focal obliteration of submucosal arterial blood flow. That is a total of 3 or 4 accessories. In contrast, multiple hemoclips (e.g., 6–8) were required to empirically close such PPIUs in this study and more HCs in this and other studies for PPIUs 20 mm or larger [4–8]. In addition, DEP monitoring reduced the number of patients who needed colonoscopic treatment by 22.9%, because if no arterial blood flow was detected in the PPIU by DEP, this predicted that DPPIUH would not occur.

## Discussion

This novel study was designed to evaluate previously unreported causes and potential risk factors for development of delayed hemorrhage after colon polypectomy. We first focused on arterial blood flow detection in ulcers created by polypectomy and the correlation with artery size in the polyp resection margins of patients either with or without delayed hemorrhage. There was a high correlation between Doppler probe detection of arterial blood flow and medium- or large-sized arteries. Using bivariate and multivariable analyses, including classification tree methodology, our next focus was to evaluate these two new risk factors in conjunction with others previously reported. In the 96 patients, we analyzed 16 different potential risk factors and documented that DEP positivity in the PPIU, medium or large artery size on polyp histology, right colon location of the polyp, and empiric PPIU hemoclip closure were significant risk factors for severe DPPIUH. However, several other previously reported clinical, colonoscopic, or polyp risk factors did not increase the risk of DPPIUH nor predict prevention of it. These were gender, ethnicity, age, chronic anti-platelet or anti-coagulant drug use, ASA or Charlson risk scores, polyp or PPIU sizes (between 10 and 40 mm), polyp configuration (sessile or pedunculated), and polyp histologic type. Our last aim was to compare colonoscopic treatments for the prevention of DPPIUH (e.g., DEP guided vs. empiric HC closure), which has not been previously reported in either cohort or randomized controlled trials (RCTs). Our results were that in contrast to empiric hemoclip closure of PPIUs which was associated with an increased risk of delayed PPIUH in this high-risk cohort study, DEP monitoring and focal obliteration of arterial blood flow in PPIUs significantly decreased and prevented DPPIUH.

Empiric hemoclip closure was not the cause of delayed PPIU hemorrhage, but was associated with delayed PPIU bleeding. That is because the anatomic and physiologic basis for delayed PPIU hemorrhage is a submucosal artery with blood flow in the PPIU which can cause severe PPIUH in high-risk patients when hemoclips do not obliterate submucosal arterial blood flow, hemoclips fall off the PPIU or the PPIU is in the left colon or rectum.

What are the clinical and technical implications of these new findings and why might they interest clinical gastroenterologists and colonoscopists? First, we provide anatomic and physiologic evidence about how and why monitoring arterial blood flow in PPIUs and target treatment in the submucosa to obliterate arterial blood flow can effectively and safely prevent DPPIUH throughout the colon and rectum. This is in contrast with single-layer mucosal closure of the PPIU with hemoclips which buries submucosal arteries without identifying them, not targeting the submucosal artery for treatment, nor always obliterating arterial blood flow [13]. When the hemoclips in the mucosa slough off in a few days after empiric HC closure, submucosal arteries are exposed and can cause severe delayed bleeding. This was exemplified in the 15 high-risk patients with DPPIH in this report who were previously treated with empiric hemoclip closure. See Figs. 3 and 4. Second, prediction of the risk of DPPIUH is feasible either using the risk factors described or the classification tree shown in Fig. 5. Third, not all patients will benefit from empiric HC closure of PPIUs since this treatment is not reported to be effective in the left colon or rectum where polyps are often located. Also in our study 22.9% of patients with PPIUs between 10 and 40 mm did not have arterial blood flow detected by DEP, none were treated, and none developed DPPIUH. At the polyp resection margins, 81% of those with negative DEP monitoring had either no or small arteries, as illustrated in Fig. 4. Empiric HC closure would not have benefited any of these patients. However, it is likely that some colonoscopists without DEP monitoring would have utilized empiric HC closure as prophylaxis to prevent DPPIU in similar high-risk patients because 68.2% (15/22) were on one or more anti-thrombotic medications and the majority were in the right colon. See Table 1. Third, DEP monitoring is easy to learn and teach others how to use and results of blood flow detection are reproducible, especially for GI ulcers [14, 15, 20]. Lastly, the relative costs for ambulatory surgical centers (ASCs) and medical procedure units (MPUs) are of major interest to most practicing gastroenterologists since accessories such as hemoclips are expensive and not currently reimbursed in the US via current procedural terminology (CPT) codes.

There are limitations in our study. First, this was a prospective cohort study but was not a RCT to compare head-to-head empiric HC closure with DEP-guided treatment for the prevention of DPPIUH. This study was an observational, cohort study and not an RCT. Treatments were not randomized and this may have confounded results and may limit interpretations about the comparisons of DEP-guided treatment and empiric clip closure to prevent delayed PPIU hemorrhage. Nevertheless, our analysis allowed us to compare anatomic results, risks, and DPPIUH rates of these two treatments in high-risk patients. Further insight about risk factors of delayed PPIUH was gained by the multivariable analysis. Second, some readers may question the generalizability of our study because of the types of patients selected and potential bias. This was a study of the vascular anatomy of polyps and arterial blood flow detection. Not all the patients who are treated in our MPUs were included because they either did not have DEP monitoring of blood flow, prospective follow-up to 30 days, or histologically evaluable polyps. Nor were all patients who had empiric HC closure in our institutions included because many did not have DEP monitoring prior to the prophylactic hemoclip closure. However, if DEP monitoring had been performed, those with negative arterial blood flow would not have bled nor benefited from empiric HC closure. Third, this was an observational cohort study and not a randomized controlled trial (RCT). As such, we did not collect nor are we able to provide RCT information in a CONSORT study flow diagram, including number of cases screened, number excluded, reasons for exclusion, dropouts, and other information. Fourth, we did not include subgroups of patients whose polyps were removed by either cold snare or endoscopic submucosal dissection (ESD). Further studies are highly recommended to expand results to these newer techniques. Fifth, we did not collect data on minor or less severe delayed PPIU bleeds that did not require hospitalization. These are important to document, particularly in an RCT of high-risk patients.

Sixth, because of their training and experience, some colonoscopists who routinely use empiric HC closure may be reluctant to change their management practices by adapting new approaches to prevent DPPIUH and potentially reduce costs. Seventh, our sample size while substantial was also not large enough for validation using a set aside validation dataset. Only internal cross validation was carried out. Last, results from logistic regression analysis were not included because they were less accurate and much less interpretable than the classification tree modeling. This is not surprising since it is difficult for logistic models to capture interactions or hierarchies which the classification tree models do. Therefore, multivariable logistic results are not reported.

There are major advantages to our study, the results, and implications to clinical practice for the prevention of one of the most common complications of colon polypectomy—delayed bleeding. First, we report the anatomic and physiologic cause of DPPIUH, its prediction, and a practical way to improve prophylactic treatment to prevent DPPIUH throughout the colon. The newly identified risks and predictors of DPPIUH are directly applicable to clinical practice of gastroenterologists who perform polypectomies. Second, we present a practical and easy way to reduce the risk of DPPIUH at the bedside based upon DEP monitoring of blood flow in PPIUs after polypectomy. These techniques can be easily introduced into clinical practice and training programs [20]. Third, we expanded the risk assessment to patients with PPIU’s between 10 and 19 mm. They are a large group of colonoscopy patients who have not been reported upon except in one previous negative study [21], but who are at increased risk for DPPIUH. In contrast, most previous RCTs and cohort reports of empiric HC closure only included polyps 20 mm or larger in size [4–8]. Unlike empiric HC closure which is not effective in the left colon or rectum, DEP monitoring and targeted obliteration of arterial blood flow is effective throughout the colon and rectum. Therefore, we have expanded the scope of risk, increased effectiveness in the entire colon, and potentially increased the likelihood that more colonoscopists who perform polypectomies will utilize DEP monitoring during screening or surveillance of this large subset of patients with moderate- or large-sized polyps. That is more convenient for patients and could reduce the number of them being referred to interventional endoscopists for a second colonoscopy to remove moderate or large polyps throughout the colon and also could reduce healthcare costs. However, improvements in training for both EMR and DEP monitoring for risk stratification and as a guide to focal treatment will be required in GI fellowship training programs and in other post-graduate venues to accomplish these changes [20]. Fourth, the results of this study along with a recent large negative RCT report about empiric hemoclip closure [22] have the potential of changing both current recommendations of colonoscopy experts and GI society practice guidelines about empiric HC closure and how to improve prophylactic treatment to prevent delayed PPIUH especially in the left colon and rectum. Lastly, based upon the cost of accessories, the number of hemoclips used in different treatments and the reduction in the proportion of patients who need hemoclipping when DEP monitoring is used (because no arterial blood flow is detected), we anticipate that DEP monitoring of blood flow and targeted treatment will be associated with significantly reduced direct treatment costs compared to empiric HC closure. To document this, a formal cost analysis is warranted [23–25].

Our conclusions of this study are: 1. There was a high correlation between DEP positivity in PPIUs, medium or large artery size on polyp histology, and a high risk of severe DPPIUH. 2. Significant risk factors for severe DPPIUH were DEP-positive arterial blood flow detection in PPIUs, right colon polyp location, empiric PPIU HC closure, and medium or large arteries at the polyp resection margin. 3. DEP-guided treatment effectively and safely prevented DPPIUH in contrast to empiric PPIU closure which was a risk factor for DPPIUH in high-risk patients after removal of benign polyps with 10–40 mm PPIUs throughout the colon.

## Data Availability

The data that support the findings of this study are available in the manuscript. Investigators may obtain further details by requesting these from the corresponding author.

## Abbreviations

ASA: American Society of Anesthesiology (Score)
ASC: Ambulatory surgical center
C: Concordance statistic
CA: California
CIs: 95% Confidence intervals
CPT: Current Procedural Terminology
CURE: DDRCC: Center for Ulcer Research and Education: Digestive Diseases Research Core Center
DEP: Doppler endoscopic probe
DPPIUHs: Delayed post-polypectomy-induced ulcer hemorrhages
EMR: Endoscopic mucosal resection
ESD: Endoscopic submucosal dissection
GI: Gastroenterology
HCs: Through-the-scope hemoclips
IRB: Institutional Review Board
mm: Millimeters
MPU: Medical procedures unit
MVA: Multivariable (logistic) analysis
NBVV: Non-bleeding visible vessel
PPIUs: Post-polypectomy-induced ulcers
PUB: Peptic ulcer bleeding
RCT: Randomized controlled trial
ROC: Receiver operator curve
SRH: Stigmata of recent hemorrhage
UGI: Upper gastrointestinal
